# Reporting quality of randomized controlled trials in otolaryngology: review of adherence to the CONSORT statement

**DOI:** 10.1186/s40463-018-0277-8

**Published:** 2018-05-15

**Authors:** Yu Qing Huang, Katsiaryna Traore, Badr Ibrahim, Maida J. Sewitch, Lily H. P. Nguyen

**Affiliations:** 1 Faculty of Medicine, McGill University, Montreal, Quebec Canada; 2 Department of Otolaryngology – Head and Neck Surgery, University of Montreal, Montreal, Quebec Canada; 3 Department of Otolaryngology – Head and Neck Surgery, McGill University, Montreal, Quebec Canada; 4 Division of Clinical Epidemiology, Research Institute of the McGill University Health Centre, Montreal, Quebec Canada; 5 Center for Medical Education, McGill University, Montreal, Quebec Canada; 6 Department of ORL-HNS, Montreal Children’s Hospital, 1001 Boulevard Décarie, Montréal, Quebec H4A 3J1 Canada

**Keywords:** CONSORT adherence, RCTs in otolaryngology, Reporting quality

## Abstract

**Background:**

Randomized controlled trials are the gold standard in medical and surgical research to assess the efficacy of therapeutic interventions. The reporting of these trials should be of high quality to allow readers’ appropriate interpretation and application.

**Methods:**

The objectives of our study were to assess the extent to which the recent Otolaryngology – Head and Neck Surgery (ORL-HNS) randomized control trials in the top nine journals and in the top Canadian journal comply with the Consolidated Standards of Reporting Trials (CONSORT) statement, and to identify the CONSORT items most in need of improvement. Based on the impact factor and circulation number of 2014, the top nine Otolaryngology journals and the top Canadian Otolaryngology journal were selected and were searched to identify RCTs published in English and between 2010 and 2014. Two authors independently reviewed and extracted data using a standardized data extraction form constructed with the help of a medical librarian. Our outcome was to assess the adherence of articles reporting to the CONSORT items. Descriptive statistics were used.

**Results:**

One hundred and eighty-two Otolaryngologic RCTs were identified in the top nine international journals and in the top Canadian journal. The inter-rater reliability between two raters was 0.32. The extent of adherence to CONSORT Statement ranged from 25 to 93.5% with a mean of 59.0% and a median of 59.4%. Only 6.5% of RCTs described the individual responsible for enrolling and assigning subjects and method of randomization; 32.4% reported the estimated effect size and precision; 40.6% reported a sample size calculation and 32.4% mentioned external validity or implications of the findings.

**Conclusion:**

Findings revealed that the reporting of RCTs in the top nine ORL-HNS journals and in the top Canadian ORL-HNS journal is suboptimal. The quality of reporting can be improved by addressing the three CONSORT items found most deficient in this study namely, sample size calculations, estimated effect size and precision, and external validity.

## Background

Randomized Controlled Trials (RCTs) are the preferred study design for comparing therapeutic interventions in medicine; they are considered the cornerstone of evidence-based medicine. However, poor reporting of RCTs impedes adequate understanding of the clinical indications. Readers require clear, transparent and complete information to assess the quality and results of a trial. Because biases can occur in all aspects of studies, poor reporting limits the reader’s appreciation of the result’s validity [1]. Flawed reporting that omits important methodological details further prevents their incorporation in systematic reviews and meta-analyses [2]. To improve clarity and transparency of reporting of RCTs, the Consolidated Standards of Reporting Trials (CONSORT) statement was released in 1996 and revised in 2001 and in 2010. The CONSORT Statement and the corresponding checklist summarize the essential items that should be reported.

The quality of reporting of RCTs in surgery is inferior to that in medicine [3]. Important differences in RCTs implementation exist in surgical disciplines from the medical disciplines. Challenges in fulfilling the criteria for RCTs including blinding and the creation of placebo patients have led to suboptimal quality of reporting, as demonstrated in a study across six surgical specialties [4]. In ORL-HNS, this shortcoming has been especially noted by Ah-See et al. in 1998, where they analyzed RCTs published over 30-year period (1966-1995) and concluded that the quality of reporting in the domain was unsatisfactory [5]. The CONSORT Non Pharmacological Treatments (CONSORT-NPT) was released with the goal to remediate to the poor adherence to the CONSORT checklist of RCTs in surgical specialties. Nevertheless, recent similar studies assessing the quality of reporting of RCTs in General Surgery and Plastic Surgery have revealed even poorer adherence to CONSORT-NPT compared to the standard CONSORT checklist [3, 4]. With the CONSORT checklist updated in 2010, there are great hopes that publications in surgical specialties will have improved RCTs reporting. Most recently, Peters et al. scored 18 articles published in ORL-HNS journals reporting a mean score of 71.8% with a significant lower grade to general medical journals [6]. To date, there have been no studies of a large number of RCTs investigating the compliance of ORL-HNS to the 2010 version of CONSORT checklist. Our primary outcomes were to evaluate the adherence to the CONSORT checklist during the period 2011-2014 and identify the items most in need of amelioration. Specifically, we recorded the adherence of RCTs to items of the CONSORT 2010 checklist to determine the progression in quality of reporting in ORL-HNS compared to the assessment of Ah-See et al. conducted 16 years ago [5].

## Methods

This study is exempted from institutional board review as all articles were publicly available.

### Selection of ORL-HNS journals

On a review of the highest-ranking impact factor international ORL-HNS journals of 2014 with the highest number of circulation, top nine journals were chosen, and one top Canadian otolaryngology journal was included in our study to add on the national perspective.

### Search method

With the assistance of a medical librarian at a tertiary centre, we performed a structured search of the MEDLINE database to identify all RCTs published in the top nine journals and in the top Canadian journal between January 1, 2011, and June 4, 2014, corresponding to the CONSORT 2010 update. Each title and abstract from the search resulted articles were screened for inclusion and exclusion criteria. Studies describing interventions performed on human subjects and written in English language were included. Exclusion criteria were animal studies, reviews, and non-RCTs. All references satisfying the inclusion criteria were further screened to ensure that the study fulfilled our search requirements.

### Rater training

All included articles were read in-depth either by the first or the second author. To assure inter-observer concordance, the reviewers (YQH and KT) were trained to first score separately the same five RCTs. Both their results were compared and verified by another senior reviewer (BI). Then, ten randomly selected studies were evaluated separately by all three reviewers and, compared with the results of an epidemiologist (MJS). Meanings and interpretations of all CONSORT criteria were discussed, and the consensus was reached among reviewers where discrepancies existed. Finally, the two reviewers (YQH and KT) each read exhaustively and scored each independently half of the remaining RCTs sorted by alphabetical order of the first author’s name.

#### Statistical analyses

Descriptive statistics consisting of frequencies and percentages calculations were used to portray characteristics of our series.

## Results

**Fig. 1:**
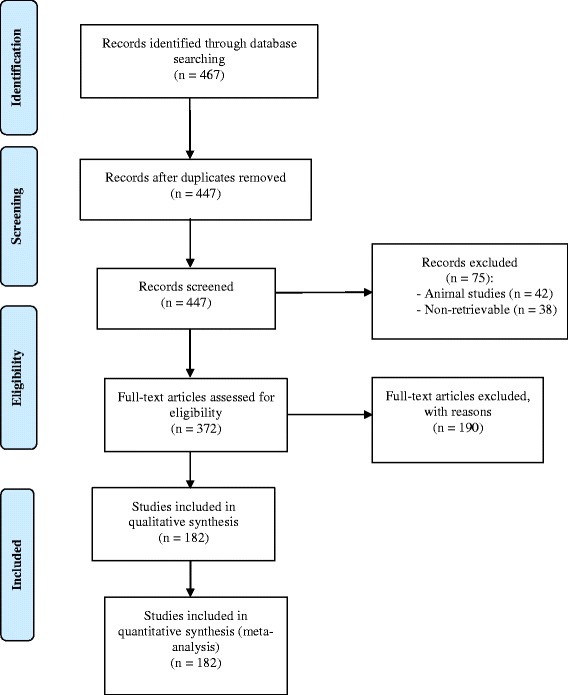
Flow Diagram of Article Selections

The inter-rater reliability between the two raters (YQH and KT) was 0.32 using Cohen’s Kappa with observed agreement of 0.87. The total number of RCTs identified by title or abstract was 467 (Fig. 1). Twenty of the articles were duplicates, and 265 did not meet inclusion criteria. The remaining 182 RCTs which came from eight different journals (Table 1) were read in full by either YQH or KT. Most articles were found in the *Otolaryngology - Head and Neck Surgery* (25.8% of all trials) and in the *Laryngoscope* (32.3% of all trials).

**Table 1:** Selected Journals in ORL-HNS

Journal Name (Country)	
*Laryngoscope*^a^(American)	
*Archives of Otolaryngology-Head and Neck Surgery* *=JAMA Otolaryngology - Head And Neck Surgery*^a^(American)	
*Annals of Otology, Rhinology and Laryngology*(American)	
*Otolaryngology - Head and Neck Surgery*^a^(American)	
*Current Opinion In Otolaryngology And Head And Neck Surgery* (British)	
*Journal of Otolaryngology - Head and Neck Surgery* (Canadian)	
*American Journal of Otolaryngology* (American)	
*Otolaryngologic Clinics of North America* (American)	
*Journal of Laryngology and Otology* (British)	
*Clinical Otolaryngology* (British)^a^	

^a^ Journals endorsing CONSORT http://www.consort-statement.org/about-consort/endorsers

### Extent of adherence to the CONSORT statement

The extent of adherence to the CONSORT Statement is the percentage of CONSORT items reported in the article. The mean extent of adherence found for 182 RCTs included in our study was 59.0%, with a range 25 to 93.5% and a median of 59.4%.

### CONSORT checklist items

To assess the quality of reporting, the most recent version of the CONSORT Statement was used. All CONSORT items and the frequency of adherence to the individual criterion are shown in Table 2. The items that were described in more than 90% of articles are the report of eligibility criteria of participants (92.3%), of intervention (99.5%), of used statistical methods (96.7%), of number of participants throughout different steps of the study (94.0%), of included patients number (90.1%), and of interpretation (96.2%). The items that were described in less than 50% of trials are the reporting of primary and secondary outcomes (42.3%), sample size calculation (40.6%), person in charge of randomization, allocation and assignment of participants (6.6%), effect size (32.4%), and generalizability (32.4%).

**Table 2:** Frequency and Percentage of Adherence to Individual Criterion of the CONSORT 2010 Checklist

Criterion	CONSORT item	Frequency	%
1a	Identification as a randomized trial in the title	80	44.0%
1b	Structured summary of trial design, methods, results, and conclusions	5	2.7%
2a	Scientific background and explanation of rationale	182	100%
2b	Specific objectives or hypotheses	182	100%
3a	Description of trial design (such as parallel, factorial) including allocation ratio	182	100%
3b	Important changes to methods after trial commencement (such as eligibility criteria), with reasons	3	100%
4a	Eligibility criteria for participants	168	92.3%
4b	Settings and locations where the data were collected	140	76.9
5	The interventions for each group with sufficient details to allow replication, including how and when they were actually administered	181	99.5%
6a	Completely defined pre-specified primary and secondary outcome measures, including how and when they were assessed	77	42.3%
6b	Any changes to trial outcomes after the trial commenced, with reasons	1	100%
7a	How sample size was determined	74	40.6%
7b	When applicable, explanation of any interim analyses and stopping guidelines	2	100%
8a	Method used to generate the random allocation sequence	106	58.6%
8b	Type of randomization; details of any restriction (such as blocking and block size)	45	24.7%
9	Mechanism used to implement the random allocation sequence (such as sequentially numbered containers), describing any steps taken to conceal the sequence until interventions were assigned	113	62.1%
10	Who generated the random allocation sequence, who enrolled participants, and who assigned participants to interventions	12	6.6%
11a	If done, who was blinded after assignment to interventions (for example, participants, care providers, those assessing outcomes) and how	108	59.7%
11b	If relevant, description of the similarity of interventions	54	32.3%
12a	Statistical methods used to compare groups for primary and secondary outcomes	176	96.7%
12b	Methods for additional analyses, such as subgroup analyses and adjusted analyses	23	88.5%
13a	For each group, the numbers of participants who were randomly assigned, received intended treatment, and were analyzed for the primary outcome	171	94.0%
13b	For each group, losses and exclusions after randomization, together with reasons	146	80.2%
14a	Dates defining the periods of recruitment and follow-up	96	52.7%
14b	Why the trial ended or was stopped	1	100%
15	A table showing baseline demographic and clinical characteristics for each group	132	72.5%
16	For each group, number of participants (denominator) included in each analysis and whether the analysis was by original assigned groups	164	90.1%
17a	For each primary and secondary outcome, results for each group, and the estimated effect size and its precision (such as 95% confidence interval)	59	32.4%
17b	For binary outcomes, presentation of both absolute and relative effect sizes is recommended	13	41.9%
18	Results of any other analyses performed, including subgroup analyses and adjusted analyses, distinguishing pre-specified from exploratory	7	6.5%
19	All important harms or unintended effects in each group (for specific guidance see CONSORT for harms)	138	78.0%
20	Trial limitations, addressing sources of potential bias, imprecision, and, if relevant, multiplicity of analyses	109	59.9%
21	Generalizability (external validity, applicability) of the trial findings	59	32.4%
22	Interpretation consistent with results, balancing benefits and harms, and considering other relevant evidence	175	96.2%
23	Registration number and name of trial registry	19	10.4%
24	Where the full trial protocol can be accessed, if available	7	3.8%
25	Sources of funding and other support (such as supply of drugs), role of funders	156	85.7%

Adapted from www.consort-statement.org

## Discussion

Our study revealed an overall mean adherence to the CONSORT 2010 checklist of 59.0% in a total of 182 surgical RCTs published in the top nine ORL-HNS journals and in the top Canadian ORL-HNS. No article satisfied all criteria evaluated in the study. To our knowledge, this is the first study to review a very large number of RCTs to assess the compliance of ORL-HNS literature in all its subspecialities to the 2010 CONSORT checklist.

Our results are comparable to those published by Ah-See et al. in 1998. They showed a mean score of 7.3/12 criteria (60.8%) in 295 articles analyzed from a scoring system derived from the CONSORT Statement published in 1996. The adherence rate in the present study (59.0%), reflects the dearth of improvement in RCT reporting in ORL-HNS literature, despite multiple important updates on CONSORT guidelines since its first publication. Ifeacho et al. found that RCTs relating to adenotonsillectomy in ORL-HNS had substandard quality of reporting with 51-60% adherence to CONSORT using the 2010 checklist [7]. Carlton et al., by using the same checklist, evaluated 38 RCTs on surgical procedures in head and neck oncology surgery and found a mean checklist score of 45.4% [8]. Our results support and widen the applicability of Ifeacho et al. and of Carlton et al. findings by expanding them to the entire scope of ORL-HNS literature by including all its subspecialities and thus having a larger search return.

By looking at the scores of individual items in the CONSORT 2010 checklist, we observe that the criteria pertaining to the background section are reported. A hundred percent of trials contained scientific background, specific objectives and description. Furthermore, items that were best reported in the present study were elements from abstracts. Authors are more careful and unlikely to miss key elements of abstracts as they represent the most condensed form of the message conveyed by a study. Contrastingly, the full paper provides the opportunity to address more in depth essential details that clinicians may not feel comfortable addressing (e.g. sample size calculation) and hence do not report them precisely. Interestingly, however, Knobloch et al. have found that abstract reporting of surgical RCTs has also been plagued by suboptimal adherence to CONSORT guidelines [9]. This raises a question regarding the role that peer-reviewed journals can have in further promoting and enforcing CONSORT guidelines.

The lowest-ranking items were from the methodology, results and discussion sections. With only 40.6% of trials reporting on sample size calculations, we believe that the readership could have legitimate concerns regarding the statistical significance and robustness of the results put forward by a vast majority of RCTs examined in the present study. Indeed, sample size calculation allows the reader to independently assess and validate the power of the study. As a larger sample size provides the best guarantee to decrease both type I and type II errors it is critical for a reader to understand the process by which a certain sample size has been determined in order to adequately detect significant variations in different treatment groups. The omission may trigger questions of statistical integrity amongst readers [10, 11]. In order to improve the design and ultimately the reporting of such intricate, yet essential, parts of an RCT we recommend that a statistician and/or epidemiologist be included in the research team supervising RCTs. Diaz-Ordaz et al., as well as another study performed by our group (unpublished data) support the concept of the multidisciplinary approach to reporting RCTs. These studies have shown that the inclusion of an epidemiologist/statistician in the author list correlated with a higher propensity of reporting sample size calculations [12].

The present study demonstrates that only 32.4% of trials report the estimated effect size and precision (e.g., 95% CI). While *p*-values are often looked at for statistical significance, a full appreciation of the magnitude of the phenomenon observed is only possible by providing the effect size [13].

Lastly, our study shows that 32.4% of included studies reported on external validity. This information is key for readers to evaluate the applicability of the trial’s results in the reader’s respective context. Evidence suggests that quality of reporting correlates with better quality in the conduct of the trial [14]. Nevertheless, achieving full marks on the CONSORT statement does not guarantee high quality or clinical relevance.

Our study has several limitations. First, the included RCTs were divided in two and were assessed independently by each reviewer. However, we included a training period where inter-rater variability was proven to be quasi-null and a third reviewer in equivocal cases. Generalizability of the findings may be limited as articles were written in the English language and found in the MEDLINE database. We acknowledge, more importantly, the large breadth of otolaryngology publications outside of otolaryngology journals, rendering our study ineligible to reflect the reporting quality of the entire publishing otolaryngology community. Indeed, we are aware that our findings only present the reporting quality of RCTs selected based on restricting criteria (10 Otolaryngologic journals, specific period of time).

## Conclusion

In conclusion, this study shows that the quality of reporting of RCTs in the top nine ORL-HNS journals and in the top Canadian journal can be improved, specifically in the areas of methodology, results and discussions of papers. The CONSORT statement promotes standardization of reporting of trials in the literature and should be adopted by authors for writing reports of trials. Its use should be encouraged by journal editors and peer reviewers. More transparent trial reports lead to improved critical appraisal and high-quality evidence-based medicine in ORL-HNS.

## Appendix 1

### Medline Search Strategy

1. laryngoscope.jn,nj,jw,nw.
2. Archives of Otolaryngology.jn,nj,jw,nw.
3. (Annals of Otology Rhinology and Laryngology).jn,nj,jw,nw.
4. (Otolaryngology - Head and Neck Surgery).jn,nj,jw,nw.
5. (Current Opinion in Otolaryngology and Head and Neck Surgery).jn,nj,jw,nw.
6. (Journal of Otolaryngology - Head and Neck Surgery).jn,nj,jw,nw.
7. American Journal of Otolaryngology.jn,nj,jw,nw.
8. Otolaryngologic Clinics of North America.jn,nj,jw,nw.
9. (The Journal of Laryngology and Otology).jn,nj,jw,nw.
10. Clinical Otolaryngology.jn,nj,jw,nw.
11. (Journal of Laryngology and Otology).jn,nj,jw,nw.
12. 1 or 2 or 3 or 4 or 5 or 6 or 7 or 8 or 9 or 10 or 11
13. Randomized Controlled Trials as Topic/
14. randomized controlled trial/
15. Random Allocation/
16. Double Blind Method/
17. Single Blind Method/
18. clinical trial/
19. randomized controlled trial.pt.
20. RCT.mp.
21. (randomly allocated or randomized controlled trial).mp.
22. (allocated adj2 random$).mp.
23. 13 or 14 or 15 or 16 or 17 or 18 or 19 or 21 or 22
24. 12 and 23
25. limit 24 to yr. = “2011 -Current

## Abbreviations

CONSORT: Consolidated Standards of Reporting Trials
CONSORT-NPT: Consolidated Standards of Reporting Trials - Non Pharmacological Treatments
ORL-HNS: Otolaryngology Head and Neck Surgery
RCTs: Randomized Controlled Trials
